# Autologous minced cartilage implantation for unstable osteochondritis dissecans of the elbow demonstrates favourable clinical and imaging outcomes at a mean 2‐year follow‐up

**DOI:** 10.1002/jeo2.70707

**Published:** 2026-04-06

**Authors:** Tim Leschinger, Tamara Babasiz, Sebastian Wegmann, Felix Krane, Lars P. Müller, Nadine Ott

**Affiliations:** ^1^ Department of Orthopaedic and Trauma Surgery, University of Cologne Faculty of Medicine and University Hospital Cologne Cologne Germany

**Keywords:** arthroscopy, cartilage lesions, elbow joint, minced cartilage, return to sports

## Abstract

**Purpose:**

The minced cartilage technique is a single‐stage cartilage repair option established for focal cartilage defects in larger joints, but evidence for its use in the elbow remains limited. The purpose of this study was to evaluate the clinical, sports‐related and imaging outcomes of the minced cartilage technique in patients with unstable elbow osteochondritis dissecans (OCD).

**Methods:**

Patients treated with minced cartilage for elbow OCD (DiPaola Grade III–IV) were included. Clinical outcomes were assessed using the Mayo Elbow Performance Score (MEPS), Quick Disabilities of the Arm, Shoulder and Hand score (qDASH), Subjective Elbow Score (SES), range of motion (ROM), pain intensity measured by the Numeric Rating Scale and return to sports. Postoperative magnetic resonance imaging (MRI) was evaluated using the Magnetic Resonance Observation of Cartilage Repair Tissue 2.0 (MOCART 2.0) score.

**Results:**

Twelve patients with a mean age of 19.1 ± 8.1 years were included at a mean follow‐up of 2.0 ± 0.9 years. Functional outcomes were excellent, with a mean MEPS of 97.1 ± 2.6, qDASH of 6.0 ± 3.7 and SES of 95.2 ± 5.6. All patients returned to sports after a mean of 5.3 months and to their pre‐injury performance level after 6.7 months. ROM was preserved, with significant improvement in extension deficit. MRI demonstrated progressive cartilage repair over time, with no correlation between imaging and clinical outcomes. First MRI at a mean of 4.5 months showed a MOCART score of 68.6 ± 2.9, while the second MRI at a mean of 11.6 months demonstrated a significant increase to 74.0 ± 1.0 (*p* = 0.008).

**Conclusion:**

The minced cartilage technique for unstable elbow osteochondrosis dissecans resulted in excellent functional outcomes, rapid return to sports, preserved ROM and satisfactory cartilage repair on MRI imaging. These findings support its feasibility in young, athletic patients, although larger prospective studies are required.

**Level of Evidence:**

Level IV.

## Graphical abstract

Autologous minced cartilage implantation for unstable elbow osteochondrosis dissecans demonstrates favourable mid‐term clinical and magnetic resonance imaging outcomes with high return‐to‐sport rates and low complications.

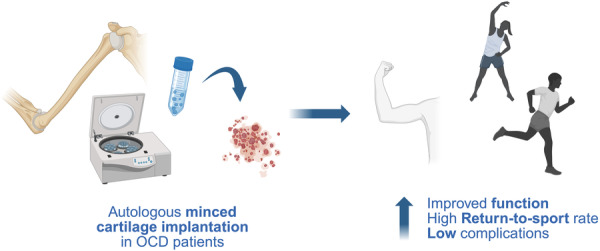

**Abbreviations**
IQR: interquartile range
MCI: minced cartilage implantation
MEPS: Mayo Elbow Performance Score
MOCART 2.0: Magnetic Resonance Observation of Cartilage Repair Tissue
MRI: magnetic resonance imaging
NRS: Numeric Rating Scale
OCD: osteochondrosis dissecans
qDASH: Quick Disabilities of the Arm, Shoulder and Hand score
ROM: range of motion
SES: Subjective Elbow Score

## INTRODUCTION

Osteochondral lesions of the elbow predominantly affect young and physically active patients and are most commonly caused by osteochondritis dissecans (OCD) of the capitellum humeri. Repetitive axial loading and valgus stress, particularly in overhead and weight‐bearing sports, are considered key contributing factors [9, 26]. Clinically, patients typically present with load‐dependent pain, mechanical symptoms, loss of motion and progressive limitation of sports participation. In advanced stages, lesion instability or fragment detachment may occur, potentially leading to early degenerative joint changes [13].

Treatment of elbow OCD is primarily guided by lesion stability and symptom severity. While stable lesions may be managed nonoperatively, unstable lesions or those associated with loose bodies usually require surgical treatment [5, 9]. According to the DiPaola classification, OCD is graded from Stage I–IV based on magnetic resonance imaging (MRI) findings. Early stages are characterized by intact articular cartilage and stable lesions, whereas advanced stages show cartilage disruption with partial or complete detachment of the osteochondral fragment, including the presence of a loose body, and are therefore considered unstable lesions [2, 8]. Commonly applied surgical techniques include fragment fixation, microfracture/bone marrow stimulation, osteochondral autograft transfer and osteochondral allograft transplantation [5]. Although these procedures can provide satisfactory clinical outcomes, they are associated with relevant limitations, including donor‐site morbidity, variable long‐term results with incomplete functional recovery, technical complexity, prolonged rehabilitation and restricted postoperative load tolerance [16, 18].

Minced cartilage implantation (MCI) is a single‐stage biological cartilage repair technique that uses autologous cartilage tissue harvested intraoperatively from the defect or detached osteochondral fragments. The cartilage is mechanically fragmented and reimplanted into a prepared defect bed with stable margins, where it is secured using biological adhesives such as fibrin and thrombin. The technique relies on the viability of chondrocytes within the cartilage fragments and avoids donor‐site morbidity and staged cell‐based procedures [10, 32]. Experimental and clinical studies in larger joints, particularly the knee and ankle, have demonstrated favourable functional outcomes and satisfactory cartilage repair on MRI [21, 22, 29].

Despite these encouraging findings, evidence regarding the use of MCI in the elbow joint remains limited. The elbow differs substantially from weight‐bearing joints in terms of cartilage thickness, joint congruency and biomechanical loading patterns, which may influence cartilage repair and integration [19]. At present, little is known about functional recovery, return to sports and structural cartilage regeneration following MCI in patients with elbow OCD.

The purpose of this study was therefore to evaluate the clinical, sports‐related and imaging outcomes of MCI in patients with unstable OCD of the elbow. We hypothesized that this technique would result in good functional outcomes, preserved range of motion (ROM) and satisfactory cartilage repair on MRI.

## METHODS

### Study design and patient selection

**Figure 1:**
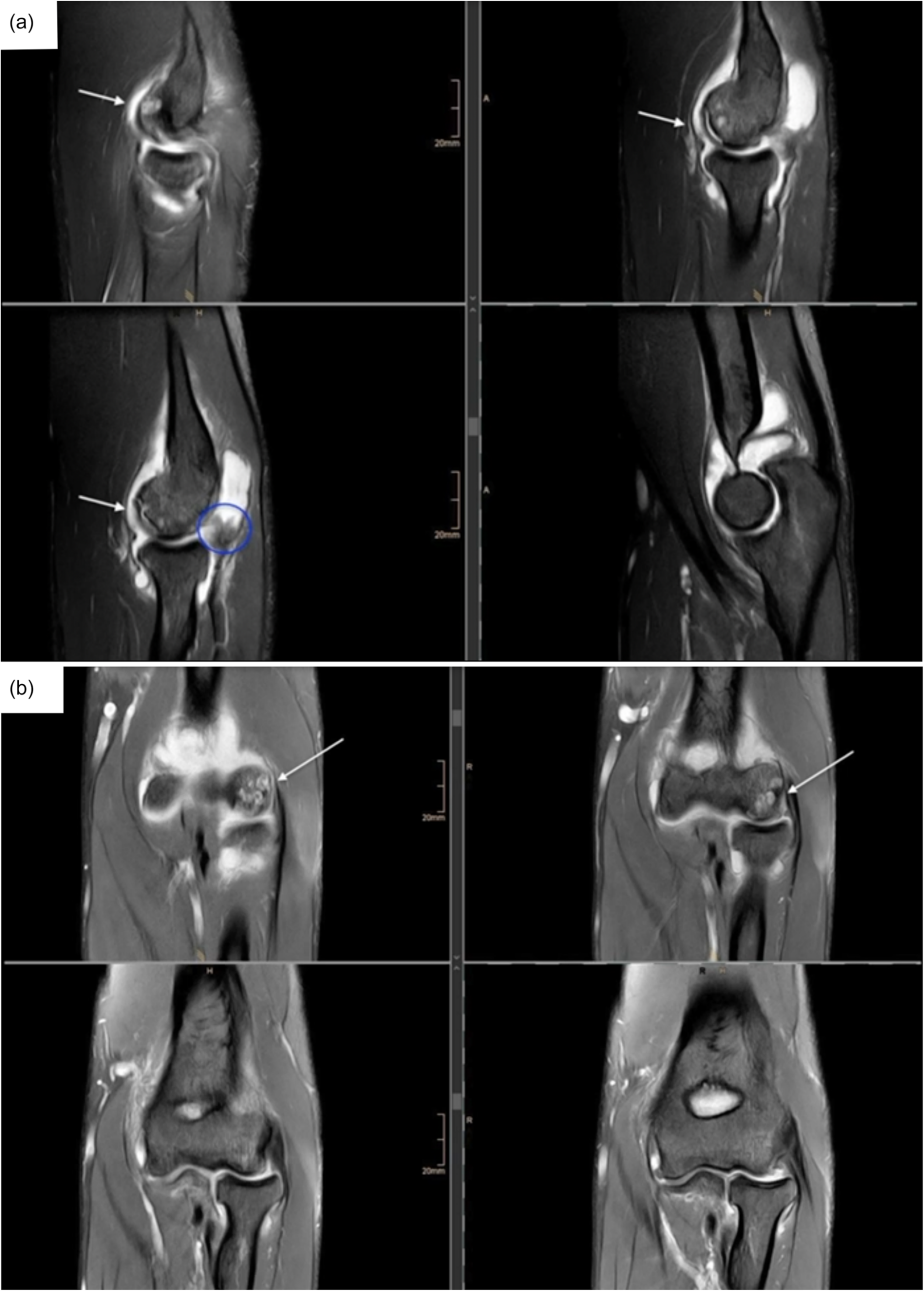
Preoperative elbow MRI of a representative patient with osteochondrosis dissecans. Representative preoperative MRI of the elbow in the sagittal (a) and coronar plane (b) demonstrating a focal osteochondral defect of the capitellum, marked by an arrow. In addition, hypertrophic tissue is visible in the region of the posterolateral plica, highlighted by the blue circle. MRI, magnetic resonance imaging.

This study was conducted as a single‐centre retrospective study. Patients who underwent a minced cartilage procedure at the elbow joint at our institution between Decemeber 2021 and November 2024 were prospectively enroled in an institutional database, and the collected data were retrospectively analysed for the present study. MCI was indicated for focal osteochondral lesions of the capitellum humeri or radial head with intact surrounding cartilage, including in situ or detached fragments and loose bodies (DiPaola Grades III–IV), as illustrated by a representative preoperative MRI (Figure 1). Contraindications include concomitant or corresponding cartilage lesions, bilateral osteochondral defects, insufficient autologous cartilage tissue for implantation and the need for concomitant procedures. Only patients who were treated with the minced cartilage technique and had a minimum clinical follow‐up of 12 months were included in the final analysis. Patients with acute traumatic cartilage lesions or those requiring additional surgical procedures, such as ligament reconstruction or fracture fixation, were excluded. The study was performed in accordance with the principles of the Declaration of Helsinki and was approved by the local ethics committee of the University of Cologne (registration number: 21‐1290).

### Surgical technique

**Figure 2:**
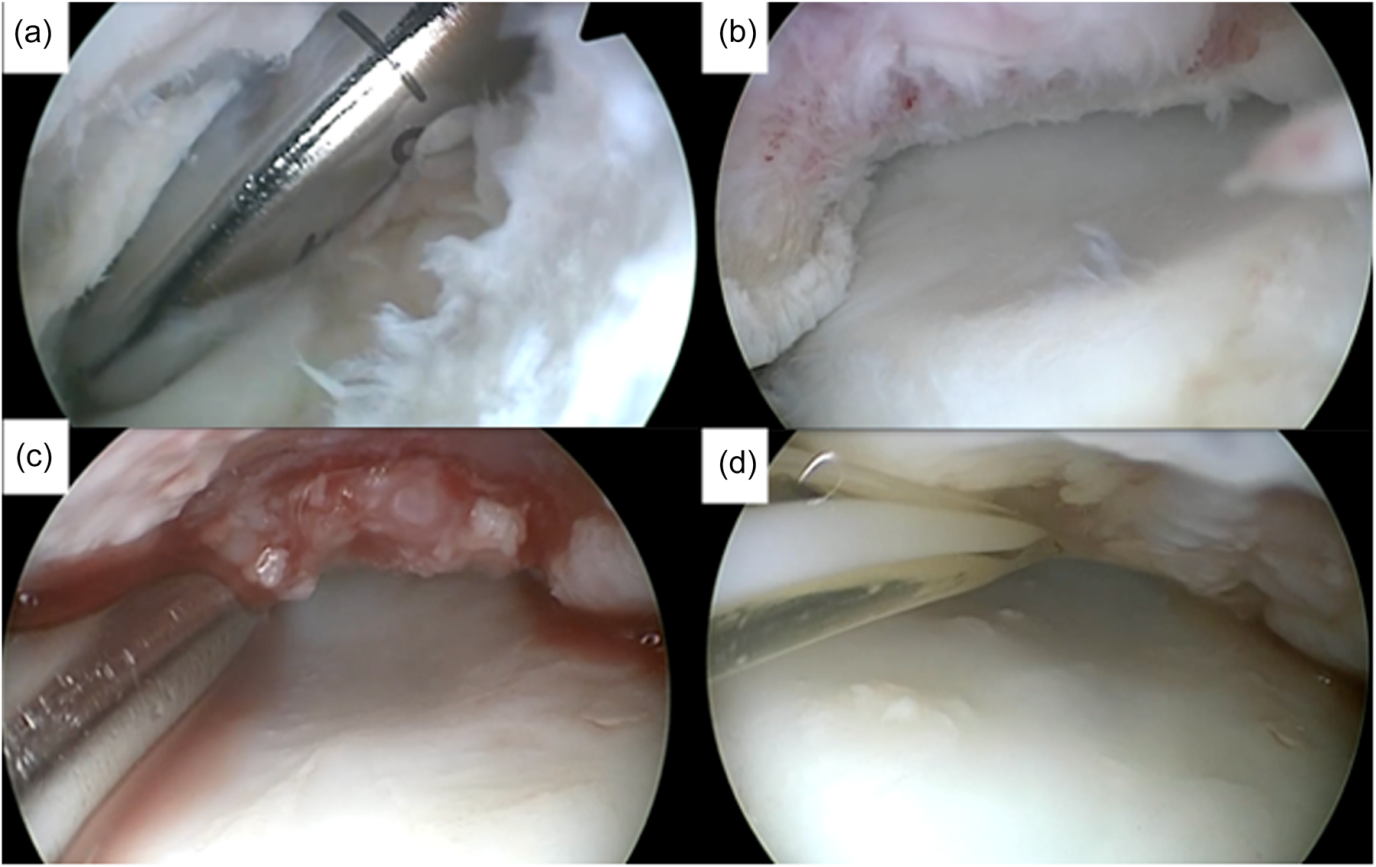
Arthroscopic intraoperative visualization of the minced cartilage implantation procedure. After visual inspection and probing of the osteochondral lesion, refixation of the fragment was deemed not feasible. Autologous cartilage tissue was subsequently harvested using a shaver introduced through the anterolateral portal, carefully releasing the osteochondral defect while preserving the integrity of the subchondral bone (a). This step enabled preparation of the cartilage fragments outside the defect site. The defect is shown from an anterolateral viewing perspective (b). The harvested cartilage particles were then mixed with autologous platelet‐rich plasma (PRP) and applied to the defect using a dedicated application cannula. The fragments were gently moulded into the defect with a blunt elevator, ensuring even defect filling without overfilling the defect margins (c). The construct was subsequently augmented with an additional layer of PRP and sealed using a thrombin‐containing mixture. Following completion of the implantation, the repair construct demonstrated sufficient stability during gentle passive range‐of‐motion testing of the elbow (d).

Elbow arthroscopy was performed with the patient in the lateral decubitus position under general anaesthesia and tourniquet control. Standard anterolateral, anteromedial and posterolateral portals were established to allow comprehensive inspection of the joint and adequate access to the lesion site. Elbow arthroscopy was initiated with a systematic inspection of the joint to identify associated intra‐articular abnormalities and to verify the indication for cartilage repair. After completion of the diagnostic phase, the arthroscope was repositioned through a posterolateral viewing portal, and an additional working portal was established under direct visualization through the soft spot. The cartilage lesion was then prepared by removing unstable tissue and clearly defining the defect borders. Any intra‐articular loose bodies encountered were extracted. Detached or partially detached cartilage tissue was subsequently processed using a motorized shaver system connected to a dedicated collection device (GraftNet Autologous Tissue Collector; Arthrex), allowing controlled fragmentation of the cartilage under continuous suction. The defect bed was further prepared by careful curettage to achieve stable surrounding cartilage, followed by thorough drying of the joint. The harvested cartilage fragments were mechanically minced to obtain particles of approximately 1–2 mm in size and combined with autologous conditioned plasma to facilitate adhesion to a carrier membrane. This construct was introduced arthroscopically into the joint and positioned within the defect using a cannula and evenly distributed to achieve complete filling without overfilling. Final fixation was achieved by application of fibrin and thrombin to ensure stable defect coverage [19] (Figure 2). After fixation, the stability of the repair tissue was assessed under direct arthroscopic visualization during gentle passive range‐of‐motion testing. All procedures were performed by two experienced elbow surgeons using a standardized surgical technique.

### Postoperative management

After surgery, the elbow was immobilized using an intraoperatively dorsally applied cast in 90° flexion in the elbow joint for 24 h. After removal of the splint, patients were allowed to begin unrestricted range‐of‐motion exercises, and no further immobilization or brace was used. Mechanical loading of the elbow was avoided for 6 weeks postoperatively. Gradual return to sports activities was permitted after 3 months, depending on individual clinical progress.

### Clinical outcome assessment

Clinical outcomes were assessed at final follow‐up using validated outcome measures, including the Mayo Elbow Performance Score (MEPS), the Quick Disabilities of the Arm, Shoulder and Hand score (qDASH), the Subjective Elbow Score (SES) and the ROM [1, 15, 23, 31]. Moreover, pain intensity was evaluated using the Numeric Rating Scale (NRS). Information on sports participation, type of sport and return to sports or high‐performance activity was documented.

### Imaging evaluation

Postoperative MRI was obtained as part of routine clinical follow‐up. All patients received at least one postoperative MRI examination, while a subset of patients underwent a second follow‐up MRI. Structural repair of the cartilage defect was assessed using the Magnetic Resonance Observation of Cartilage Repair Tissue (MOCART 2.0) score. Scores ≥80 points were categorized as excellent or near‐complete morphological repair, scores between 60 and 79 points as good repair, 40–59 points as moderate repair and scores <40 points as poor repair [4]. MRI assessments were performed independently by a trauma surgeon and a radiologist. Disagreements were resolved by consultation with a third senior trauma surgeon.

### Statistical analysis

Statistical analyses were performed using GraphPad Prism (GraphPad Software) and Microsoft Excel (Microsoft Corporation). Normality of continuous variables was assessed using the Shapiro–Wilk test. Continuous variables were assessed descriptively and are reported as mean ± standard deviation or as median with interquartile range (IQR), depending on data distribution and statistical testing. Preoperative and postoperative ROM parameters (extension deficit, flexion, pronation and supination) were compared using the Wilcoxon signed‐rank test for paired samples. Changes in MRI‐based cartilage repair quality between the first and second postoperative MRI examinations were analysed using the Wilcoxon signed‐rank test. Correlations between MRI outcomes (MOCART 2.0 score) and clinical outcome measures (MEPS, qDASH, SES), as well as patient age, were assessed using Spearman's rank correlation coefficient. Correlation results are reported as Spearman's rho with 95% confidence intervals (CIs). A *p* value < 0.05 was considered statistically significant.

## RESULTS

**Figure 3:**
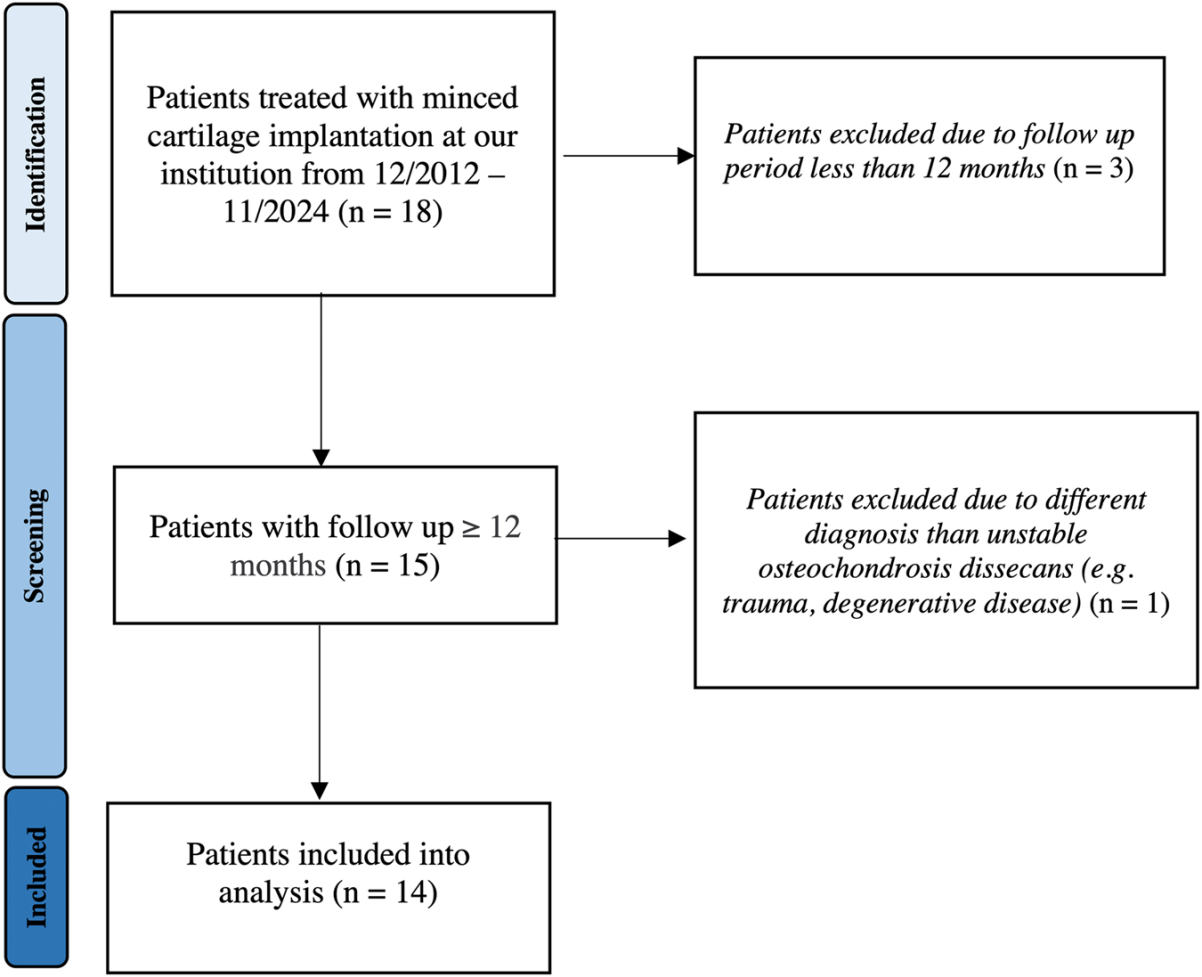
Patient flowchart of study inclusion and exclusion. Flowchart illustrating patient selection, exclusion criteria and final study cohort. During the study period (December 2021–November 2024), 18 patients underwent minced cartilage implantation. Four patients were excluded due to insufficient follow‐up or different diagnoses, resulting in a final study population of 14 patients.

Twelve patients were included in the final analysis (Figure 3). The mean age at surgery was 19.1 ± 8.1 years (range: 13–29 years). All patients presented with unstable OCD of the elbow, including five Grade III and seven Grade IV lesions. All lesions were located at the capitellum, with seven lateral and five medial lesions. No free intra‐articular loose bodies or detached fragments were observed at the time of surgery, and none of the patients presented with an associated fracture. Lesions with a surface area of approximately ≤200 mm^2^ were considered suitable for MCI technique. Preoperatively, all patients reported load‐dependent elbow pain persisting for several months to years, accompanied by restriction of sports participation and, in some cases, mechanical symptoms such as joint locking. The mean follow‐up duration was 2.0 ± 0.9 years (range: 1.05–3.89 years).

**Figure 4:**
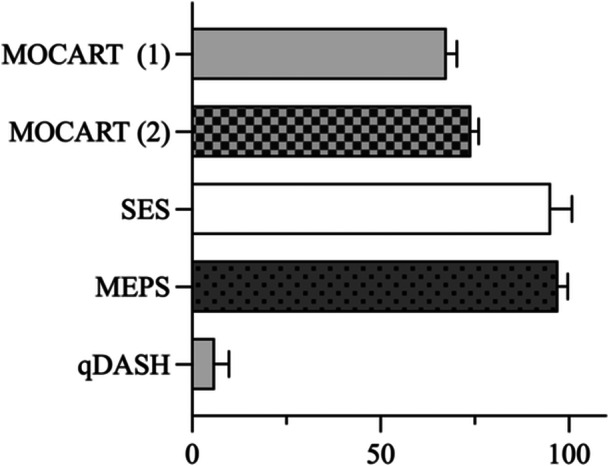
Clinical outcome scores and MRI‐based cartilage repair assessment at follow‐up. Bars represent mean values with error bars indicating standard deviation. MOCART (1) refers to the MOCART score at the first postoperative MRI follow‐up (*n* = 11), whereas MOCART (2) indicates the MOCART score at the second postoperative MRI follow‐up (*n* = 4). MEPS, Mayo Elbow Performance Score; MOCART, Magnetic Resonance Observation of Cartilage Repair Tissue; MRI, magnetic resonance imaging; qDASH, Quick Disabilities of the Arm, Shoulder and Hand score assessed at final follow‐up; SES, Subjective Elbow Score.

At final follow‐up, functional outcomes were excellent. The mean MEPS was 97.1 ± 2.6 points, the mean QuickDASH score was 6.0 ± 3.7 points, and the mean SES was 95.2 ± 5.6 points. Pain levels were minimal, with a mean NRS score of 0.83 ± 0.83 (Figure 4).

Regarding elbow ROM, the median extension deficit improved significantly from 0° (IQR 0°–15°) preoperatively to 0° (IQR 0°–0°) at final follow‐up (*p* = 0.039). Median elbow flexion remained unchanged, measuring 130° (IQR 120°–130°) preoperatively and 130° (IQR 130°–130°) at final follow‐up (*p* = 0.364). Similarly, median pronation showed no statistically significant change from 75° (IQR 70°–80°) to 80° (IQR 80°–80°; *p* = 0.344), and median supination remained stable at 80° (IQR 70°–80° preoperatively vs. 80° (IQR 80°–80°) postoperatively; *p* = 0.334). Overall, elbow ROM was preserved postoperatively, with no clinically relevant loss of motion observed at final follow‐up.

Return to sports was achieved after a mean of 5.3 ± 1.6 months. Seven of twelve patients returned to competitive sports, including three high‐performance athletes. Among these patients, four had Grade III lesions and three had Grade IV lesions according to the DiPaola classification. All patients were able to return to their preoperative level of sports participation after a mean of 6.7 ± 1.2 months, and two patients exceeded their preoperative performance level. The spectrum of sports included CrossFit, fitness training, boxing, football, handball, BMX, hockey and golf.

All patients underwent a first postoperative MRI examination at a mean of 4.5 ± 2.6 months after surgery (*n* = 11), demonstrating a mean MOCART 2.0 score of 68.6 ± 2.9. A second postoperative MRI examination was available in four patients at a mean of 11.6 ± 2.2 months postoperatively and showed a significant improvement in cartilage repair tissue quality, with a mean MOCART score of 74.0 ± 1.0 (*p *= 0.008, Figure 4).

Correlation analyses revealed no significant association between MRI‐based cartilage repair quality and clinical outcome measures. No correlation was observed between the MOCART score and the MEPS (Spearman's *ρ* = 0.06, 95% CI −0.58 to 0.65; *p* = 0.89). Similarly, no significant correlations were found between the MOCART score and the qDASH score (*ρ* = −0.24, 95% CI −0.74 to 0.44; *p* = 0.48) or the SES (*ρ* = −0.05, 95% CI −0.64 to 0.58; *p* = 0.88). Patient age was also not significantly correlated with the MOCART score (*ρ* = −0.24, 95% CI −0.75 to 0.43; *p* = 0.46).

With regard to complications, no reoperations were required, and no clinically relevant complications were observed. Three patients reported persistent crepitus without pain or functional impairment, including one patient with an asymptomatic recurrent intra‐articular loose body.

## DISCUSSION

To the best of the author's knowledge, this is the first study to report clinical and imaging outcomes of MCI in the elbow joint with a mean follow‐up of 2 years. The key finding of this study is that MCI resulted in satisfactory mid‐term clinical outcomes and a reliable return to sports in young patients with unstable elbow OCD. At a mean follow‐up of 2.0 ± 0.9 years, patients demonstrated very high functional scores, preserved ROM and a rapid return to pre‐injury sports performance. These results suggest that MCI represents a feasible surgical option for the treatment of advanced elbow OCD in a physically active patient population.

MCI has been investigated in several clinical and imaging follow‐up studies in larger joints, particularly the knee and the ankle, where favourable functional outcomes, return to sports [13, 27, 30] and satisfactory cartilage repair on MRI have been reported [14, 24, 25, 29]. In contrast, data on the application of this single‐stage biological repair technique in the elbow joint remains scarce. To date, no evidence is available regarding its clinical performance, return‐to‐sport capability and structural cartilage regeneration in patients with elbow OD. In this cohort, uniformly favourable functional outcomes were observed, with all patients achieving return to sports at an equal or improved performance level compared with the preoperative status. These findings compare favourably with reported outcomes of established surgical techniques for OCD of the humeral capitellum. A recent meta‐analysis including 24 studies reported return‐to‐sport rates of 64% following fragment fixation and 71% after debridement and marrow stimulation procedures [30]. In contrast, all patients in the present cohort were able to return to sports following MCI (100%). While direct comparisons are limited by differences in study design and patient populations, the similarly timed follow‐up and the high return‐to‐sport rate observed in this study suggest that MCI may represent a valuable alternative for the treatment of unstable elbow OCD.

Postoperative MRI findings demonstrated consistently good structural cartilage repair in all patients, with high MOCART 2.0 scores already present at the first postoperative examination and maintained during follow‐up. Notably, MOCART values in this cohort predominantly fell within a range that has previously been associated with favourable clinical outcomes. Prior studies assessing cartilage repair in the tibiofemoral joint have shown that MOCART 2.0 scores of ≥60 points are generally linked to good patient‐reported outcomes, whereas substantially higher scores are rarely achieved even after successful cartilage repair. Against this background, the MOCART results observed in the present study indicate satisfactory and clinically relevant structural repair [11, 17]. Correlation analyses revealed no significant association between MRI‐based cartilage repair quality and clinical outcome scores. This finding is consistent with previous reports in cartilage repair literature, where early or mid‐term MRI appearance does not necessarily correlate with patient‐reported outcomes or functional performance [6, 28]. Hereby, the reduced number of patients undergoing a second postoperative MRI represents a limitation of the imaging analysis. This was primarily attributable to loss to imaging follow‐up and patient‐related factors, including the absence of symptoms and perceived lack of necessity for further imaging. Importantly, the absence of a correlation between imaging and clinical outcomes highlights that clinical improvement and functional recovery can precede complete structural restoration on MRI. From a clinical perspective, patient‐reported outcomes and return to sports are highly relevant endpoints, particularly in young and athletic patients, and may not necessarily require perfect morphological cartilage appearance. Furthermore, no association was identified between patient age and MRI‐based cartilage repair quality, indicating that structural cartilage healing following MCI was not age‐dependent within the studied cohort and that comparable imaging outcomes were achieved across a broad age range.

Regarding complications following MCI, three patients reported persistent crepitus without pain or functional limitation, while one of them developed a recurrent loose body [3, 20]. When comparing the minced cartilage technique with well‐established surgical techniques for the treatment of unstable OCD of the elbow, particularly osteochondral autograft transfer systems (OATS), the low complication rate observed in this cohort appears notable. Although the OATS procedure shows favourable long‐term clinical outcomes reported in multiple studies [3, 20], it has been associated with donor‐site morbidity at the knee, including persistent pain, cartilage damage and functional impairment, with reported donor‐site complication rates ranging from 5% to over 20% depending on graft size and harvest technique [7, 12]. In addition, graft mismatch, technical complexity and prolonged rehabilitation have been reported as relevant limitations of osteochondral grafting procedures in the elbow [32]. Other commonly used techniques, such as fragment fixation or microfracture, have also been associated with variable complication profiles, including residual mechanical symptoms, incomplete defect healing and limited durability of fibrocartilaginous repair tissue [30]. In contrast, MCI avoids donor‐site morbidity, does not require graft harvesting from a secondary joint and can be performed as a single‐stage procedure. These characteristics may contribute to the low rate of clinically relevant complications observed in the present cohort. Nevertheless, direct comparative studies are required to more definitively assess complication rates and long‐term safety relative to other treatment modalities.

Compared with isolated loose body removal and debridement, MCI aims to restore the articular surface and provide biological repair tissue at the defect site. This may contribute to improved joint congruency, reduced mechanical symptoms and enhanced load distribution, potentially facilitating earlier functional recovery and return to sports. In addition, the single‐stage nature of the procedure and the absence of donor‐site morbidity may allow for a more rapid rehabilitation protocol. However, MCI is technically more demanding and requires meticulous defect preparation and stable fixation, which may prolong operative time. Furthermore, early rehabilitation must balance joint mobilization and protection of the repair tissue, which may limit aggressive early loading compared with simple debridement. Therefore, while MCI may offer potential biological advantages, its superiority regarding early return to sports remains to be confirmed in comparative studies.

Several limitations of this study must be acknowledged. The retrospective design and small sample size warrant confirmation of these findings in larger cohorts. The lack of standardized preoperative clinical scores represents a limitation of this study. Furthermore, the follow‐up duration, although exceeding 2 years on average, does not allow assessment of long‐term durability or prevention of degenerative joint changes. MRI follow‐up was incomplete, limiting the interpretation of structural repair outcomes inherent to the retrospective study design. Finally, although follow‐up exceeded 2 years on average, longer‐term outcomes and durability of the repair tissue remain unknown. Future prospective, controlled studies with larger cohorts and standardized imaging protocols are warranted to confirm these findings.

Despite these limitations, this study provides novel clinical data on the application of MCI of the elbow joint. The consistent functional improvement, rapid return to sports, preserved ROM and progressive MRI findings support the use of this single‐stage biological repair technique in young and athletic patients with unstable elbow OCD. Future prospective studies with larger cohorts, longer follow‐up and comparative designs are required to further define the role of MCI relative to established treatment options.

## CONCLUSION

The minced cartilage technique for unstable elbow OCD resulted in excellent functional outcomes, preserved ROM, rapid return to sports and satisfactory cartilage repair on MRI imaging. These findings support the feasibility of this technique in the elbow joint, particularly in young, athletic patients. Further prospective studies with larger cohorts are required to confirm long‐term outcomes.

## AUTHOR CONTRIBUTIONS

All authors contributed to the conception and design of the study. Material preparation, data collection and analysis were performed by the authors. The first draft of the manuscript was written by the authors, and all authors commented on previous versions of the manuscript. All authors read and approved the final manuscript.

## CONFLICT OF INTEREST STATEMENT

The authors declare no conflicts of interest.

## ETHICS STATEMENT

The study was approved by the local ethics committee of the University of Cologne (registration number: 21‐1290_3). Written informed consent was obtained from all patients or their legal guardians prior to participation in the study.

## Data Availability

The datasets generated during and/or analysed during the current study are available from the corresponding author on request at any time.
